# Derivation and validation of the first web-based nomogram to predict the spontaneous pregnancy after reproductive surgery using machine learning models

**DOI:** 10.3389/fendo.2024.1378157

**Published:** 2024-07-02

**Authors:** Zhenteng Liu, Meimei Wang, Shunzhi He, Xinrong Wang, Xuemei Liu, Xiaoshi Xie, Hongchu Bao

**Affiliations:** ^1^ Department of Reproductive Medicine, Yantai Yuhuangding Hospital Affiliated to Qingdao University, Yantai, Shandong, China; ^2^ Department of Reproductive Medicine, Linyi People’s Hospital, Linyi, Shandong, China; ^3^ Shandong Provincial Key Medical and Health Laboratory of Reproductive Health and Genetics (Yantai Yuhuangding Hospital), Yantai, Shandong, China

**Keywords:** reproductive surgery, spontaneous pregnancy, predictive model, online nomogram, individualized medicine, machine learning

## Abstract

**Objective:**

Infertility remains a significant global burden over the years. Reproductive surgery is an effective strategy for infertile women. Early prediction of spontaneous pregnancy after reproductive surgery is of high interest for the patients seeking the infertility treatment. However, there are no high-quality models and clinical applicable tools to predict the probability of natural conception after reproductive surgery.

**Methods:**

The eligible data involving 1013 patients who operated for infertility between June 2016 and June 2021 in Yantai Yuhuangding Hospital in China, were randomly divided into training and internal testing cohorts. 195 subjects from the Linyi People’s Hospital in China were considered for external validation. Both univariate combining with multivariate logistic regression and the least absolute shrinkage and selection operator (LASSO) algorithm were performed to identify independent predictors. Multiple common machine learning algorithms, namely logistic regression, decision tree, random forest, support vector machine, k-nearest neighbor, and extreme gradient boosting, were employed to construct the predictive models. The optimal model was verified by evaluating the model performance in both the internal and external validation datasets.

**Results:**

Six clinical indicators, including female age, infertility type, duration of infertility, intraoperative diagnosis, ovulation monitoring, and anti-Müllerian hormone (AMH) level, were screened out. Based on the logistic regression model’s superior clinical predictive value, as indicated by the area under the receiver operating characteristic curve (AUC) in both the internal (0.870) and external (0.880) validation sets, we ultimately selected it as the optimal model. Consequently, we utilized it to generate a web-based nomogram for predicting the probability of spontaneous pregnancy after reproductive surgery. Furthermore, the calibration curve, Hosmer–Lemeshow (H-L) test, the decision curve analysis (DCA) and clinical impact curve analysis (CIC) demonstrated that the model has superior calibration degree, clinical net benefit and generalization ability, which were confirmed by both internal and external validations.

**Conclusion:**

Overall, our developed first nomogram with online operation provides an early and accurate prediction for the probability of natural conception after reproductive surgery, which helps clinicians and infertile couples make sensible decision of choosing the mode of subsequent conception, natural or IVF, to further improve the clinical practices of infertility treatment.

## Introduction

During the last decades, the number of infertile couples caused by different etiologies has gradually increased worldwide since 1990 (1), resulting in a substantial medical and social burden. Nowadays, reproductive surgery and *in vitro* fertilization and embryo transfer (IVF-ET) are two main treatment strategies for infertility (2). Reproductive surgery is a minimally invasive technology that aims to restore the functional anatomy and accomplish fertility preservation to enhance the chance of natural or assisted pregnancy. The definition of spontaneous pregnancy refers to the process of achieving pregnancy without the use of assisted reproductive technologies or additional interventions, which is important for both spouses, such as saving time and expense and reducing the risk of low birth weight and birth defects in newborns. Compared with IVF, a successful reproductive operation could offer patients the opportunity for natural conception monthly and avoid the complications of IVF, such as ovarian hyperstimulation syndrome and multiple pregnancies (3). Even without spontaneous pregnancy after a 1~2-year postoperative period, endoscopic surgical procedures could provide comprehensive evaluations including anatomy and function of the reproductive organs to improve pregnancy outcome in subsequent IVF (4).

In the era of precision medicine, early prediction of the reproductive surgery outcomes, such as spontaneous pregnancy, is of high interest for the women seeking the infertility treatment. However, there is still lacking of a high-quality model and clinical applicable tool to predict the probability of natural conception after reproductive surgery. On the one hand, due to the heterogeneity of operational quality control, the longer learning curve of surgical skill, and the absence of verification of conception rates following surgery, the majority of available literature regarding postoperative pregnancy outcomes consists of small single-institution retrospective cohort studies. On the other hand, the assessment of women’s potential for fertility after operation primarily relies on the clinical experience generated by physicians, hence it is really difficult to give an individualized opinion since every patient has a unique situation. Some patients blindly adhere to attempt natural pregnancy after surgery, missing the golden time of IVF therapy, especially when the recurrence of endometriosis or hydrosalpinx requiring a second operation comes. Therefore, in order to make informed decisions regarding natural fertilization or IVF as early as possible, it is critical to timely anticipate the likelihood of spontaneous pregnancy after surgical reconstruction of reproductive function.

Notably, the prediction model derived from machine learning (ML) algorithms is a reliable and widely used statistical tool (5) that can consider various factors simultaneously to provide a probability of a specific outcome, especially in medicine (6). Nevertheless, as far as we know, there has been no research that has developed a forecasting model for the probability of natural pregnancy after reproductive surgery, and the key predictors are also under discussion. Hence, the objective of this research was to derivate and validate an analytical model based on multiple typical ML algorithms to ascertain the crucial clinical factors and provide an early personalized evaluation of probability of postoperative spontaneous pregnancy.

## Materials and methods

This prediction model study is reported in accordance with the Transparent Reporting of a Multivariable Prediction Model for Individual Prognosis or Diagnosis (TRIPOD) checklist (7). The study was approved by the Ethics Committees of Yantai Yuhuangding Hospital (YT2023–054) and Linyi People’s Hospital [LYRMYY (2023–04-036)]. Since this research was a retrospective cohort study, the data was made anonymous and there was no need for informed consent.

### Study population

Between June 2016 and June 2021, a total of 2049 individuals underwent surgical procedures for infertility at the Department of Reproductive Medicine, Yantai Yuhuangding Hospital, China. Data on demographic, preoperative clinical assessment, surgical procedure details, operative diagnosis and blood biochemical parameters were retrospectively collected from an electronic medical record system (Jiahe Meikang Information Technology, Beijing, China), which were utilized for the derivation and internal validation of the prediction model. In the external validation cohort, 363 infertile couples were hospitalized at the Department of Reproductive Medicine, Linyi People’s Hospital, from January 2019 to June 2021. Data about pregnancy of follow-up evaluations was recorded by phone call or review of outpatient clinic revisit records. The follow-up period was 2 years. The data is reviewed, extracted, and cross-checked by the expert clinical team, with two separate clinicians who were unaware of the recorded results conducting the verification. Any disagreements were resolved by roundtable consensus.

### Inclusion and exclusion criteria

The eligibility requirements were as follows: (1) age ≤38 years; (2) patients having an almost menstrual cycle (counting from the first day of one menstrual period to the first day of the next cycle) is 21 to 35 days and lasts from 3 to 7 days duration with volume of blood loss 50–80 ml; (3) spouse’s roughly normal semen quality; (4) couples’ normal sexual life; (5) patients obtaining at least one grossly functionally normal fallopian tube after surgery; (6) patients holding intentions to get a natural pregnancy after surgery during at least 2-year observation period. In contrast, the analysis did not include patients with a history of unsuccessful IVF and pathology requiring surgical treatment before the next IVF. Patients who converted to IVF treatment due to personal reasons within a 2-year follow-up period were excluded from this study. In addition, we excluded patients who needed for preimplantation genetic diagnosis and lacked primary measured data. All participants included in this research were of Han descent, and had no history of psychiatric or neurological illness, and no history of alcohol or drug abuse, and no recent history of smoke.

### Dependent variable

As a primary outcome, clinical pregnancy was defined as observation of one or more intrauterine gestational sacs by a transvaginal ultrasound scan during follow-up period after reproductive surgery. The pregnancies from artificial insemination and IVF were not taken into account, meanwhile the ectopic pregnancy was regarded as a failure.

### Independent variables selection and definition

Independent variables were selected based on the known clinically risk factors and availability in the electronic medical record system (Jiahe Meikang Information Technology, Beijing, China), which include: female age, body mass index (BMI, kg/m^2^, <20; 20–24.9; 25.0–29.9; ≥30.0), infertility type (primary or secondary), duration of infertility, history of previous pelvic surgery, and tubal patency test by hysterosalpingography (HSG, mild, moderate or severe altered tubal patency). Women voluntarily had a baseline serum AMH measurement by an ultrasensitive two-site ELISA (AnshLabs, Webster, TX, USA) (8) on the first day of hospitalization before surgery. In clinical terms, preoperative AMH was categorized into three grades based on the following criteria: low (≤1.2 ng/ml), normal (1.2–4.0 ng/ml), and high (≥4.0 ng/ml). The reference data for these grades are derived from previous literature sources in conjunction with our empirical generalizations (9–11). In addition, some patients experienced ovulation monitoring using transvaginal ultrasounds (≥2 times per menstrual cycle) to clearly define ovulation time after surgery in our or other clinics.

To assess the patient’s physical condition, common serum biochemical parameters were determined on the first day of hospitalization, as following: carbohydrate antigen 125 (CA125), total cholesterol (TC), triglyceride (TG), high-density lipoprotein (HDL), low-density lipoprotein (LDL), alanine aminotransferase (ALT), aspartate transaminase (AST), creatinine (Cr), fasting insulin (INS) and fasting glucose (Glu).

All included subjects had undergone diagnostic or operative laparoscopy combined with hysteroscopy routinely. According to intraoperative dominant manipulation, the main operative diagnoses were categorized into seven subgroups, as mentioned in the studies by Ban Frangez, H., et al. (3) and Premru-Srsen, T., et al. (12). These subcategories encompass diagnostic surgery, mild to moderate endometriosis, severe endometriosis, intramural fibroids, unilateral tubal factor, bilateral tubal factor and miscellaneous cases. See attached **Additional File 1**: **Supplementary Table 1** for more details.

### Screening independent risk factors

Firstly, covariates with a *P* value less than 0.2 from the univariate logistic analysis were chosen for the binary multivariate logistic regression analysis, which was used to determine which predictors independently associated with spontaneous pregnancy according to the backward stepwise selection with the Akaike information criterion (AIC). Odds ratios (OR) with 95% confidence interval (CI) were calculated.

To ensure accuracy of predictive factors selection, the least absolute shrinkage and selection operator (LASSO) analysis was also employed to identify the most significantly independent features from the training dataset (6), augmented with ten-fold cross-validation.

### Model construction

Six common machine learning algorithms, namely logistic regression, decision tree, random forest, support vector machine (SVM), k-nearest neighbor (KNN), and extreme gradient boosting (XGBoost), were utilized to construct the predictive model in the training cohort. Additionally, we assessed the robustness and generalization ability of the above predictive models by comparing their performance parameters including the area under the curve (AUC) of the receiver operating curve (ROC), accuracy, precision, sensitivity, and specificity in the internal and external validation sets.

### Evaluation and validation of the nomogram

We ultimately selected the logistic regression as the optimal model due to its superior clinical predictive value in both internal and external validation sets (refer to the Results section for more information). Subsequently, the nomogram was constructed using the findings from the analysis of multivariate logistic regression. In order to support their integration into the clinical setting, a *Shinyapp.io* application (https://www.shinyapps.io/) was utilized to create an interactive web-based dynamic nomogram.

To evaluate the nomogram’s prediction accuracy, the AUC of the ROC with the bootstrapping method was used to determine the discrimination of the proposed model (7). Further, the calibration curves were plotted to test the goodness-of-fit of the model concurrently accompanied with the Hosmer-Lemeshow test (13). The clinical usefulness of this nomogram was evaluated through decision curve analysis (DCA), which aimed to identify the prediction’s net benefit threshold. The nomogram’s clinical effective rate was evaluated using the clinical impact curve (CIC) (14). Last but not least, the sensitivity analyses were performed to assess how the prediction performance change with univariable models compared with that of our final nomogram from the perspective of AUC and DCA.

### Statistical analysis

R software (version 4.2.3, available for download https://www.rproject.org/) was utilized to perform all statistical analysis. Various specific packages such as “pROC”, “rms”, “ggplot2”, “dca”, “DynNom”, “tidyverse” and “mlr3” were employed. Descriptive statistics were used to summarize baseline characteristics. Continuous variables were presented as mean (standard-deviation). A complete randomized analysis of variance was used to compare differences among groups (Gaussian distribution) or Kruskal-Wallis rank sum test (nonnormal distribution). Categorical variables were expressed as frequency (percentage values), and differences among cohorts were determined using the chi-square (χ^2^), Fisher’s exact test or Kruskal-Wallis rank sum test, as appropriate. A 2-tailed *P* value <0.05 was considered statistically significant.

## Results

**Figure 1:**
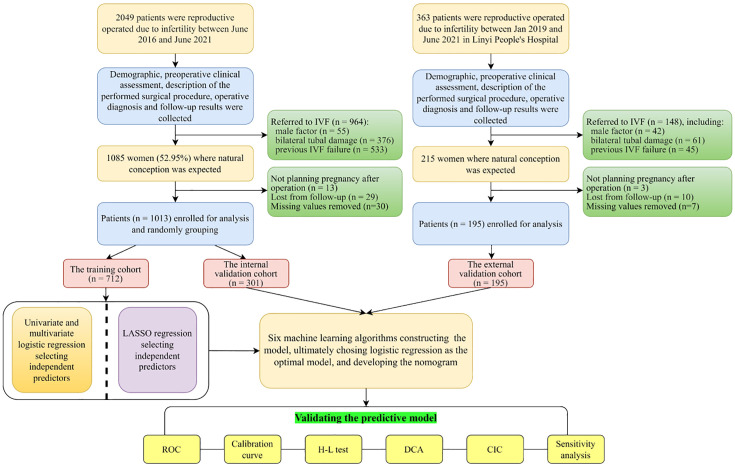
Flowchart of the study. IVF, *in vitro* fertilization; LASSO, least absolute shrinkage and selection operator; ROC, receiver operating curve; H-L test, Hosmer-Lemeshow test; DCA, decision curve analysis; CIC, clinical impact curve.

Out of 2049 operated women in Yantai Yuhuangding Hospital, 964 were immediately referred to IVF due to factors such as male infertility, damaging to bilateral fallopian tubes, or previous unsuccessful attempts at IVF. Among the remaining 1085 women, 13 ceased to plan pregnancy due to personal reasons, 29 women were lost from follow-up, and 30 subjects missed primary items, including HSG, AMH, CA125, TC, TG, INS and Glu. No significant differences were observed between the values before and after removing the missing data (**Additional File 1**: **Supplementary Table 2**). **Figure 1** displays the flowchart illustrating the process of selecting patients and designing the study.

Using a rate of 50% for the occurrence of the event in the series (spontaneous pregnancy after reproductive surgery) and considering 6 variables selected through multivariable logistic analysis, we conducted a power analysis. This analysis utilized the formula developed by Riley et al. (15), with the aim of achieving a shrinkage of predictor effects of 0.288 (pmsampsize (type = “b”, *r* squared = 0.288, parameters = 6, prevalence = 0.50) (15, 16) and obtaining a required sample size of 385 patients and 32.08 events per variable. Finally, a total of 1013 individuals in Yantai Yuhuangding Hospital were enrolled in this study to develop the model, which satisfied the minimum sample size.

Among 1013 enrolled infertile women, the percentage of women who conceived spontaneously is 51.7% (n = 524/1013) in the postoperative 2-year period. The enrolled patients were randomly divided into a training set (70.3%, n = 713) which was used to construct a model, and an internal validation set (29.7%, n = 301). Meanwhile, an additional 195 patients from Linyi People’s Hospital were utilized for external validation. The process of patient selection can be seen in **Figure 1**. No significant difference is observed in the spontaneous pregnancy rate (51.4%, 52.5% and 50.3%, *P*<0.05), clinical baseline characteristics and laboratory data among the three datasets (training, internal and external validation sets), indicating good homogeneity between the three datasets, which was summarized in **Table 1**.

**Table 1:** Baseline characteristics of all patients in the training cohort and validation cohort.

Variables	Total	Training cohort	Internal validation cohort	External validation cohort	*P*-value (overall)
	*N=1208*	*N=712*	*N=301*	*N=195*	
Pregnancy^a^:					0.886
No	586 (48.5%)	346 (48.6%)	143 (47.5%)	97 (49.7%)	
Yes	622 (51.5%)	366 (51.4%)	158 (52.5%)	98 (50.3%)	
Age (years)^b^	31.2 (3.40)	31.3 (3.41)	31.2 (3.19)	30.9 (3.72)	0.851
BMI (kg/m^2^)^a^:					0.075
20~24.9	676 (56.0%)	400 (56.2%)	169 (56.1%)	107 (54.9%)	
<20	243 (20.1%)	136 (19.1%)	68 (22.6%)	39 (20.0%)	
25~29.9	244 (20.2%)	145 (20.4%)	60 (19.9%)	39 (20.0%)	
≥30	45 (3.73%)	31 (4.35%)	4 (1.33%)	10 (5.13%)	
Infertility_type^a^:					0.269
primary	608 (50.3%)	367 (51.5%)	143 (47.5%)	98 (50.3%)	
secondary	600 (49.7%)	345 (48.5%)	158 (52.5%)	97 (49.7%)	
Duration of infertility (years)^b^	2.57 (1.35)	2.62 (1.37)	2.50 (1.33)	2.51 (1.33)	0.181
Previous_pelvic_surgery^a^:					0.868
no	1179 (97.6%)	696 (97.8%)	293 (97.3%)	190 (97.4%)	
yes	29 (2.40%)	16 (2.25%)	8 (2.66%)	5 (2.56%)	
HSG^a^:					0.067
mild	518 (42.9%)	308 (43.3%)	114 (37.9%)	96 (49.2%)	
moderate	351 (29.1%)	197 (27.7%)	105 (34.9%)	49 (25.1%)	
severe	339 (28.1%)	207 (29.1%)	82 (27.2%)	50 (25.6%)	
Surgical_procedures^a^:					0.778
diagnostic	186 (15.4%)	106 (14.9%)	44 (14.6%)	36 (18.5%)	
endometriosis_mild_moderate	235 (19.5%)	134 (18.8%)	62 (20.6%)	39 (20.0%)	
endometriosis_severe	116 (9.60%)	71 (9.97%)	29 (9.63%)	16 (8.21%)	
intramural_fibroids	24 (1.99%)	13 (1.83%)	7 (2.33%)	4 (2.05%)	
tubal_factor_unilateral	226 (18.7%)	144 (20.2%)	48 (15.9%)	34 (17.4%)	
tubal_factor_bilateral	254 (21.0%)	148 (20.8%)	65 (21.6%)	41 (21.0%)	
miscellaneous	167 (13.8%)	96 (13.5%)	46 (15.3%)	25 (12.8%)	
Ovulation_monitoring^a^:					0.398
no	489 (40.5%)	294 (41.3%)	115 (38.2%)	80 (41.0%)	
yes	719 (59.5%)	418 (58.7%)	186 (61.8%)	115 (59.0%)	
AMH^a^:					0.545
normal	1124 (93.0%)	664 (93.3%)	276 (91.7%)	184 (94.4%)	
low	31 (2.57%)	21 (2.95%)	9 (2.99%)	1 (0.51%)	
high	53 (4.39%)	27 (3.79%)	16 (5.32%)	10 (5.13%)	
CA125 (U/mL)^b^	24.3 (8.26)	24.2 (8.21)	24.6 (8.49)	24.2 (8.13)	0.430
TC (mmol/L)^b^	4.57 (0.84)	4.57 (0.85)	4.57 (0.82)	4.58 (0.83)	0.985
TG (mmol/L)^b^	1.19 (0.49)	1.20 (0.50)	1.16 (0.50)	1.21 (0.47)	0.244
HDLC (mmol/L)^b^	1.53 (0.39)	1.53 (0.39)	1.56 (0.39)	1.50 (0.37)	0.152
LDLC (mmol/L)^b^	2.59 (0.63)	2.59 (0.62)	2.58 (0.66)	2.60 (0.60)	0.757
ALT (U/L)^b^	25.6 (11.9)	25.5 (11.0)	26.2 (13.9)	25.2 (11.6)	0.427
AST (U/L)^b^	25.1 (8.45)	25.3 (8.65)	24.9 (8.26)	25.0 (8.05)	0.493
Cr (μmol/L)^b^	57.9 (10.4)	58.2 (10.2)	57.3 (10.5)	57.4 (10.7)	0.208
INS (uU/mL)^b^	13.8 (6.58)	13.8 (6.54)	13.8 (6.88)	13.9 (6.31)	0.963
Glu (mmol/L)^b^	5.04 (0.77)	5.01 (0.76)	5.12 (0.77)	5.03 (0.83)	0.052

^a^Categorical variables were expressed as frequency (percentage values), and differences among cohorts were determined using the chi-square (χ^2^), Fisher’s exact test or Kruskal-Wallis rank sum test, as appropriate. ^b^All values were mean (standard-deviation) and tested by analysis of variance (Gaussian distribution) or Kruskal-Wallis rank sum test (nonnormal distribution). BMI, body mass index; HSG, hysterosalpingography; AMH, anti-Müllerian hormone; CA125, carbohydrate antigen 125; TC, total cholesterol; TG, triglyceride; HDLC, high-density lipoprotein; LDLC, low-density lipoprotein; ALT, alanine aminotransferase; AST, aspartate transaminase; Cr, creatinine; INS, fasting insulin; Glu, fasting glucose.

### Independent risk factors

First, 19 variables were analyzed via univariate logistic analysis, and eight features with statistically significant differences (*P*<0.2) were picked out. Next, the aforementioned variables were incorporated into the original multivariate logistic regression model (AIC=614.62), as shown in **Table 2**. Finally, according to the principle of AIC minimization (AIC=610.43), six independent predictors were selected in the final logistic regression model by the backward stepwise selection. **Table 2** displays the precise coefficients for each individual factor.

**Table 2:** Univariate and multivariate logistic regression analysis to determine independent predictors associated with spontaneous pregnancy according to the backward stepwise selection with the Akaike information criterion (AIC).

Characteristics	Univariate analysis			Multivariate analysis (original) (AIC=614.62)			Multivariate analysis (final) (AIC=610.43)			
	OR	95%CI	*P* value	OR	95%CI	*P* value	β	OR	95%CI	*P* value
Age (years)	1.29	1.22–1.36	<0.001	0.79	0.74–0.85	<0.001	-0.227	0.80	0.75–0.85	<0.001
BMI (kg/m^2^)										
20~24.9	reference			reference						
<20	0.94	0.63–1.38	0.737	0.73	0.42–1.27	0.262				
25~29.9	1.16	0.80–1.70	0.435	1.06	0.63–1.78	0.837				
≥30	2.34	1.08–5.11	0.032	0.59	0.21–1.63	0.309				
Infertility_type										
primary	reference			reference			reference			
secondary	0.26	0.19–0.35	<0.001	3.06	2.01–4.66	<0.001	1.107	3.02	1.99–4.60	<0.001
Duration of infertility (years)	2.39	2.05–2.79	<0.001	0.44	0.36–0.54	<0.001	-0.807	0.45	0.37–0.54	<0.001
Previous_pelvic_surgery										
no	reference									
yes	1.37	0.50–3.72	0.537							
HSG										
mild	reference			reference						
moderate	0.73	0.51–1.05	0.088	1.53	0.91–2.58	0.105				
severe	0.85	0.60–1.21	0.359	1.50	0.92–2.45	0.105				
Surgical_procedures										
diagnostic	reference			reference			reference			
EM_mild_moderate	0.19	0.11–0.35	<0.001	7.67	3.58–16.41	<0.001	1.992	7.33	3.45–15.58	<0.001
EM_severe	3.33	1.68–6.62	0.001	0.29	0.12–0.70	0.005	-1.131	0.32	0.14–0.76	0.010
intramural_fibroids	0.4	0.12–1.37	0.143	1.31	0.25–6.85	0.751	0.454	1.58	0.30–8.20	0.590
tubal_factor_unilateral	0.58	0.35–0.97	0.038	2.05	1.05–3.98	0.035	0.700	2.01	1.04–3.89	0.037
tubal_factor_bilateral	0.99	0.6–1.64	0.984	0.96	0.51–1.82	0.906	-0.066	0.94	0.5–1.75	0.836
miscellaneous	2.54	1.40–4.59	0.002	0.22	0.09–0.51	<0.001	-1.440	0.24	0.10–0.54	0.001
Ovulation_monitoring										
no	reference			reference			reference			
yes	0.35	0.26–0.48	<0.001	2.41	1.59–3.65	<0.001	0.876	2.40	1.59–3.63	0.001
AMH										
normal	reference			reference			reference			
low	6.61	1.93–22.63	0.003	0.12	0.02–0.61	0.011	-2.183	0.11	0.02–0.56	0.008
high	0.88	0.41–1.91	0.748	5.18	1.63–16.45	0.005	1.516	4.55	1.46–14.2	0.009
CA125 (U/mL)	1.00	0.98–1.02	0.985							
TC (mmol/L)	0.9	0.75–1.07	0.215							
TG (mmol/L)	1.09	0.81–1.47	0.557							
HDL (mmol/L)	1.08	0.74–1.57	0.705							
LDL (mmol/L)	0.85	0.67–1.07	0.167							
ALT (U/L)	1.00	0.98–1.01	0.498							
AST (U/L)	1.00	0.98–1.02	0.846							
Cr (μmol/L)	0.99	0.98–1.01	0.232							
INS (uU/mL)	0.99	0.97–1.01	0.443							
Glu (mmol/L)	0.98	0.8–1.19	0.808							

AIC, Akaike information criterion; OR, odds ratio; CI, confidence interval; BMI, body mass index; HSG=, hysterosalpingography; AMH, anti-Müllerian hormone; CA125, carbohydrate antigen 125; TC, total cholesterol; TG, triglyceride; HDLC, high-density lipoprotein; LDLC, low-density lipoprotein; ALT, alanine aminotransferase; AST, aspartate transaminase; Cr, creatinine; INS, fasting insulin; Glu, fasting glucose.

**Figure 2:**
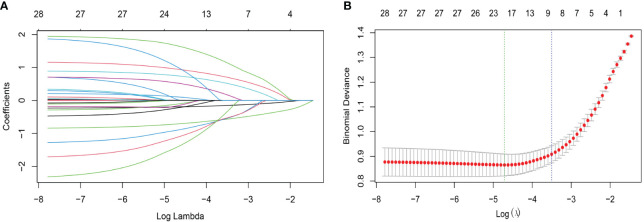
Characteristic variable screening based on the LASSO analysis with ten-fold cross-validation. **(A)** Plot of the LASSO coefficient profiles against the log (λ, lambda) sequence. **(B)** Tuning parameter (λ, lambda) selection of deviance in the LASSO regression based on the minimum criteria (left dotted line) and the 1-SE criteria (right dotted line). In the present study, predictor’s selection was according to the 1-SE criteria (right dotted line), where 9 nonzero coefficients were selected (6 predictors including dummy variables, more details are in **Additional file 1**: **Supplementary Table 3**). LASSO, least absolute shrinkage and selection operator; SE, standard error.

Regarding LASSO regression, **Supplementary Table 3** (**Additional File 1**) displays the coefficients, while **Figure 2A** illustrates a profile of the coefficients. Significantly, the optimal tuning parameter for LASSO regression, denoted as “Lambda (λ)”, was determined to be 0.036 at the point where the partial likelihood binomial deviance achieved its lowest value (refer to **Figure 2A**). As shown in **Figure 2B**, six predictors including dummy variables were independently associated with non-zero coefficients within one standard error of the log λ minimum in the LASSO analysis. These variables were selected for the most regularized and parsimonious model.

Encouragingly, both the number and name of the final independent factors (age, infertility type, duration of infertility, surgical procedures, ovulation monitoring and AMH) were good concordance between multivariate logistic analysis and LASSO algorithm, indicating that the above selected factors were appropriate.

### Clinical predictive value of the machine learning models

**Figure 3:**
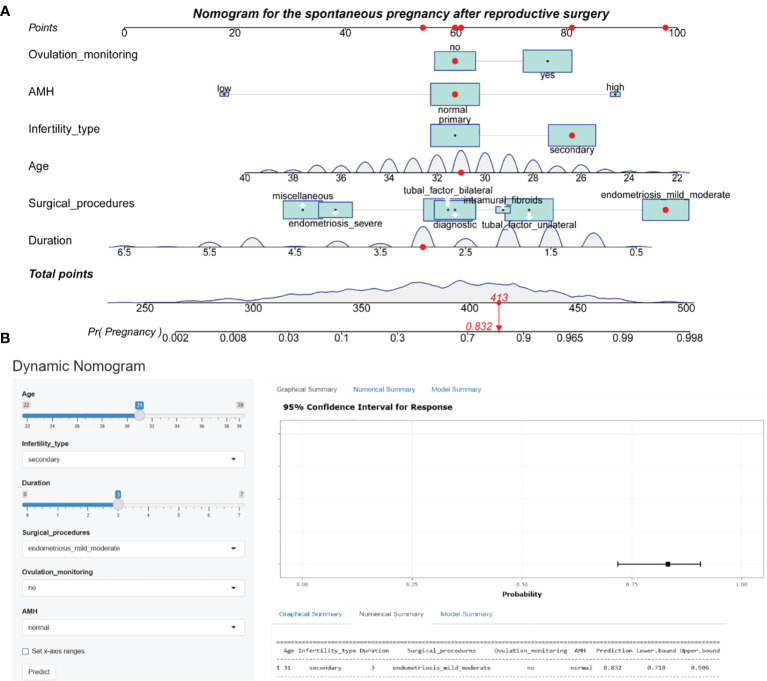
Nomogram prediction model for the spontaneous pregnancy after reproductive surgery. **(A)** Established nomogram in the training cohort by incorporating the following six parameters: age, infertility type, duration of infertility, main surgical procedures, ovulation monitoring and AMH. **(B)** Corresponding web-based dynamic nomogram accessible at https://yyyzhentengliu.shinyapps.io/DynNomforSPRafterRS/. AMH, anti-Müllerian hormone.

Hyperparameters were further optimized for each model to ensure best performance. In the training set, bootstrapping method with 1000 resamples was used to assess the performance of the models. Initially, as shown in **Table 3**, random forest exhibited superior performance, with an AUR of 0.902 (95% CI 0.888–0.912), followed by logistic regression, with an AUR of 0.892 (95% CI: 0.870–0.915) in the training set. However, the logistic regression model performed the best among all models in terms of AUC across both internal and external validations. Therefore, from the perspective of the model interpretability and stability, the logistic regression model is chosen as our final model. Consequently, the individualized predictive nomogram (**Figure 3A**) and an interactive user-friendly online calculator (**Figure 3B**) were established (https://yyyzhentengliu.shinyapps.io/DynNomforSPRafterRS/). For example, when an infertile woman is aged 31 years old, and the duration of the secondary infertility is 3 years with a normal AMH level, suffering from mild to moderate endometriosis, without ovulation monitoring using transvaginal ultrasounds (≥2 times per menstrual cycle) after surgery, we could impute that her probability of receiving natural conception after surgery during 2-year period is 83.2% (**Figures 3A, B**).

**Table 3:** Performance parameters of the 6 machine learning prediction models in the training, internal and external validation sets.

Predictive models	AUC	Accuracy	Precision	Sensitivity	Specificity
Training set					
Logistic regression	**0.892**	0.784	0.788	0.758	0.806
Decision tree	0.815	0.748	0.757	0.713	0.785
Random forest	0.902	0.792	0.789	0.734	0.809
SVM	0.807	0.727	0.699	0.775	0.679
KNN	0.787	0.711	0.749	0.613	0.803
XGBoost	0.858	0.770	0.773	0.739	0.798
Internal validation					
Logistic regression	**0.870**	0.782	0.786	0.741	0.829
Decision tree	0.812	0.742	0.742	0.711	0.782
Random forest	0.868	0.782	0.784	0.737	0.825
SVM	0.806	0.744	0.701	0.804	0.680
KNN	0.784	0.681	0.691	0.594	0.759
XGBoost	0.857	0.768	0.772	0.738	0.795
External validation					
Logistic regression	**0.880**	0.810	0.849	0.752	0.867
Decision tree	0.802	0.722	0.729	0.702	0.685
Random forest	0.879	0.793	0.787	0.747	0.855
SVM	0.804	0.742	0.698	0.805	0.682
KNN	0.786	0.682	0.694	0.598	0.762
XGBoost	0.861	0.771	0.774	0.739	0.798

AUC, area under the receiver operating characteristic curve; SVM, support vector machine; KNN, k-nearest neighbor; XGBoost, extreme gradient boosting.

### Model validation of discrimination and calibration

**Figure 4:**
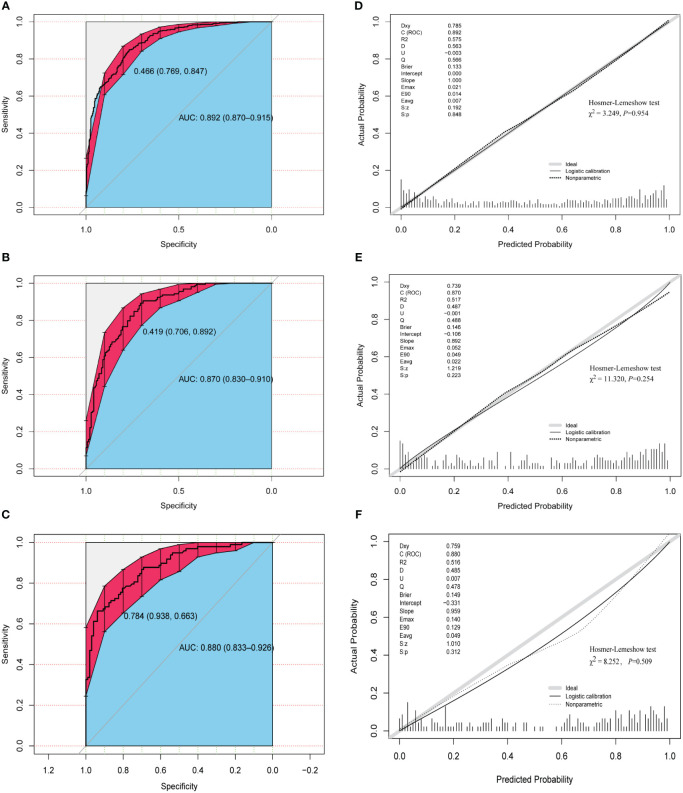
Assessment of discrimination and calibration of the model. ROC and AUC using the bootstrap method (resampling = 1000) of the nomogram prediction model in the training cohort **(A)**, internal test cohort **(B)**, and external test cohort **(C)**. The dotted vertical lines represent the 95% confidence interval. The calibration curves and Hosmer-Lemeshow test of the nomogram prediction model for the training cohort **(D)**, internal test cohort **(E)**, and external test cohort **(F)**. ROC, receiver operating characteristic; AUC, area under the ROC curve.

**Figures 4A–C** demonstrate that the final model had an AUC of 0.892 (95% CI 0.870–0.915) in the training group. In the internal and external validation groups, the AUC was 0.870 (95% CI 0.830–0.910) and 0.880 (95% CI 0.833–0.926) respectively, indicating good predictive ability in discrimination between pregnancy negative and positive cases.

The three calibration curves of this model were fairly similar to the ideal curve (**Figures 4D–F**), suggesting that the estimated outcomes aligned with the real observations. In addition, Hosmer–Lemeshow test indicated that all *P*-values of the model are greater than 0.05 in the three cohorts (**Figures 4D–F**), suggesting that there was no statistical fit-departure between the predicted and observed values.

### Clinical utility of the predictive model

**Figure 5:**
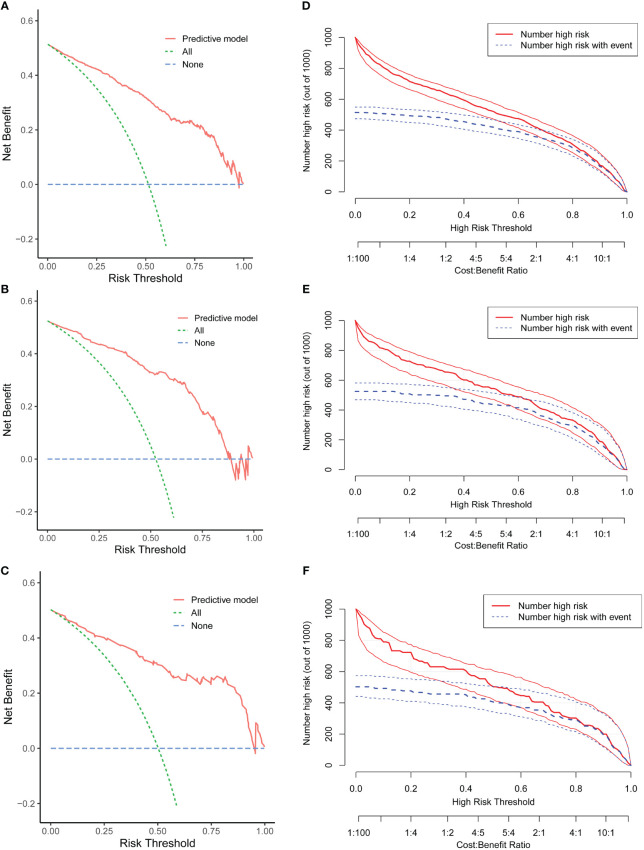
Evaluation of the clinical utility of the nomogram. Decision curve analysis (DCA) of the training cohort **(A)**, internal test cohort **(B)**, and external test cohort **(C)**. Clinical impact curve (CIC) of the training cohort **(D)**, internal test cohort **(E)**, and external test cohort **(F)**.

The DCA revealed that the clinical prediction guided by the nomogram leads to better net benefits and more extensive range of cutoff probabilities in detecting spontaneous pregnancy than either the treat-all scheme or the treat-none scheme in the three datasets (**Figures 5A–C**, **Additional File 1**: **Supplementary Table 4** displays net benefits for various threshold probabilities).

Concurrently, the CIC demonstrated remarkable predictive accuracy of this nomogram in predicting spontaneous conception, exhibiting greater efficacy in differentiating patients within the high and low probability categories in the training set (**Figure 5D**) and validation groups (**Figures 5E, F**).

### Sensitivity analyses

**Figure 6:**
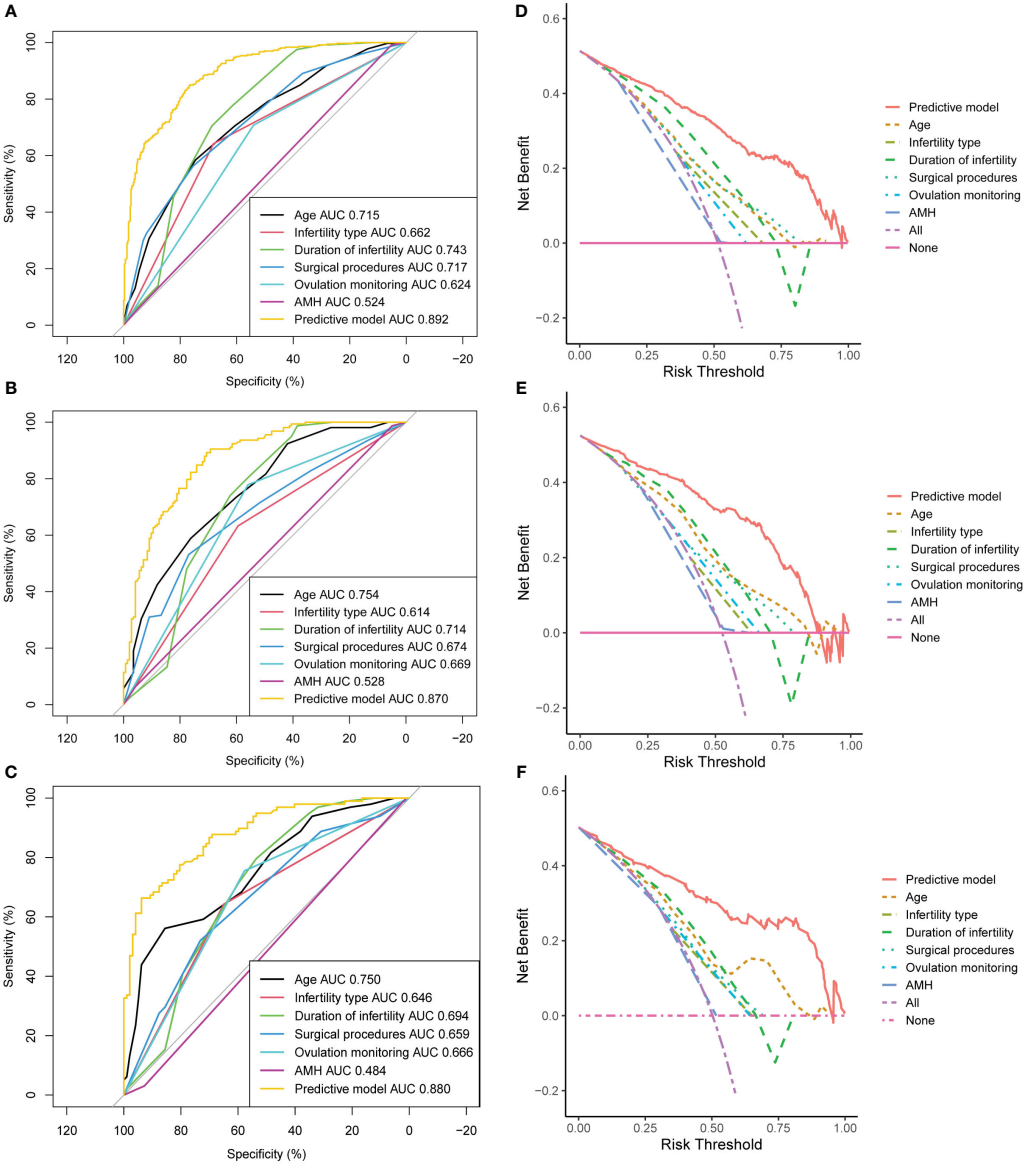
Sensitivity analysis of the model. Area under the ROC curve (AUC) of the training cohort **(A)**, internal test cohort **(B)**, and external test cohort **(C)**. Decision curve analysis (DCA) of the training cohort **(D)**, internal test cohort **(E)**, and external test cohort **(F)**. AMH, anti-Müllerian hormone.

AUC values of single independent predictors (female age, infertility type, duration of infertility, surgical procedures, ovulation monitoring and AMH) were significantly lower than that of the predictive nomogram (**Figures 6A–C**). These trends were also observed in DCA, i.e., our developed nomogram had the highest net benefit within a range of threshold compared with any of the univariate models (**Figures 6D–F**).

## Discussion

Benefiting from the technological innovations of recent years and the popularization of standard reproductive surgical procedures, reproductive surgery is widely considered one of the major therapeutic schedules for infertility, even though its significance was once doubted a few years ago (17). Counseling inevitably arises in clinical practice regarding the chance of pregnancy once reproduction function is reconstructed. However, a reliable prediction model has not been reported so far. Our current study developed the first publicly free nomogram that integrates key clinical features (patient age, infertility type, duration of infertility, intraoperative diagnosis, ovulation using ultrasound monitoring and serum AMH level) to impute the likelihood of spontaneous pregnancy following reproductive surgery. Notably, the model demonstrated superior discriminative power, good calibration and clinical utility, which were confirmed by both internal and external validations.

It has been well established that woman’s age was strongly associated with conceiving success after reproductive surgery (18) and/or IVF (19). For every extra year of female age during their childbearing years, the pregnancy rate decreases by around 20% (OR = 0.80, *P <*0.001) according to our findings. In addition, women experiencing secondary infertility have a three-fold higher likelihood of achieving a spontaneous pregnancy (OR = 3.02, *P <*0.001) in comparison to those with primary infertility. The above findings were a bit higher than those reported by Ban Frangez, H., et al. (3). The effect of age on conception rate after surgery may be related to ovarian function, because age directly influences ovarian reserve, embryos quality and endometrial receptivity. In terms of infertility type, it is likely that the chance of pregnancy in this secondary cohort of women is higher as they have previously proven to be fertile. Additionally, in our study, duration of subfertility is an independent factor to predict natural conception after surgery, which is in accordance with a recent study (4). The possible explanation is that the longer the years of infertility, the longer the underlying pathologies (salpingitis, hydrosalpinx, pelvic adhesions, and endometriosis, etc.) could persist, causing a greater difficulty of the surgical operation, which limits the therapeutic effect. Another possible reason is that women with a longer infertility duration tend to be older.

AMH immunoassays are widely accepted for assessing ovarian reserve and guiding the personalized ovulation induction regimen in IVF (20, 21). Previous studies indicated that there was an independent correlation between AMH and live birth among women undergoing IVF (22). However, little attention has been paid to the significance of AMH level in predicting natural conception following infertility surgery. In our institution, patients are generally willing to accept the serum AMH detection to assess self-ovarian reserve before surgical treatment. In the present study, it was found that anti-Müllerian hormone (AMH) exhibited an independent predictive value for spontaneous conception following reproductive surgery, leading to its inclusion in the prognostic model. Hence, it is crucial to take into account not only the surgical interventions but also to devote adequate attention to the precise evaluation of ovarian function reserve when predicting the surgical outcomes. Nevertheless, antral follicle count (AFC) and follicle-stimulating hormone level were not tested in most of the patients in the present study, so we could not evaluate the relationship between the two and natural conception.

Another interesting finding in this study was that using transvaginal ultrasound scan to aid in detecting ovulation in our or other clinics significantly improves patients’ pregnancy outcomes (OR = 2.40, *P* = 0.001). Attempts to commence natural gestation as early as possible after surgery, how to accurately judge the day of ovulation is very important. The transvaginal sonogram is widely acknowledged as one of the most convenient and accurate techniques for identifying ovulation. In this study, the decision of detecting ovulation or not is mostly based on patient preference. Our data support that patients even with almost regular menstruation should be further assessed for the fertile window in the menstrual cycle after surgery to guide the opportunity of couple’s sex life.

Among the three currently most frequently used separate endometriosis classification/scoring systems (i.e. revised American Society for Reproductive Medicine (rASRM), Enzian and Endometriosis Fertility Index (EFI)), the EFI is the only widely recognized to have significant predictive value for natural or IVF conception after surgery for patients affected by endometriosis (23, 24). Nevertheless, several limitations should be noted. On the one hand, the EFI solely relies on the macroscopic assessment of the present condition of the fallopian tubes and ovaries, without considering the biomarker function of ovarian reserve like AMH or AFC. On the other hand, the EFI system does not provide any information to predict pregnancy achievement for non-endometriosis patients. Our model not only has some overlapped features with EFI, such as the fertility history (female age, type and years of infertility, rASRM score), but also combines the ovulation monitoring and ovarian reserve information, which would be a useful addition to the EFI to some extent. Moreover, this model basically covers most common etiologies of surgically amenable infertility.

Previous studies demonstrated that the existence of subserous or intraligamentary fibroids and nonmalignant ovarian cysts have no well-defined impact on fertility (3, 25). Due to the limited sample size observed in these diagnoses, we opted to merged the above subgroup with those patients without pronounced pathological changes at laparoscopy to the diagnostic laparoscopy group (**Additional File 1**: **Supplementary Table 1**). No significant association was detected between BMI and natural conception after surgical management, which is in line with the previous papers (3, 4). In addition, our multivariate logistic analysis indicates that HSG is not suitable as an independent predictive factor for pregnancy outcome. The reason may be related to the confounding (often low) image quality and the subjectivity of the observer. Therefore, clinical physicians inferring the patient’s prognosis should not be formulated based on HSG status alone but should synthetically consider other key factors. Another interesting negative finding was that the probability of natural pregnancy after surgery in women with intramural fibroids (*P*=0.158) or bilateral tubal lesions (*P*=0.836) was comparable to the diagnostic laparoscopy group. When normal anatomy was confirmed at laparoscopy, the patients are termed the unexplained infertility, which has been proven to be more difficult to deal with, even in IVF. Furthermore, for ones suffering from clear driving factors of infertility (mild/moderate endometriosis or unilateral tubal factor), laparoscopy can significantly improve fertility in these patients by correcting anatomical fallopian tubal abnormalities, and destroying concurrent endometriosis lesions, as shown by our results (**Table 2**). However, severe pathologies (stage III-IV endometriosis or miscellaneous) would inevitably lead to a lower prognosis, even though at least one roughly functionally normal fallopian tube was retained.

In our center, all surgery was carried out by the same professional reproductive surgery team which has been established for about 20 years, and an average of 400 laparoscopic procedures combined with hysteroscopy are performed annually. This can be attributed to the extensive training, meticulous procedures like fimbriae eversion with sutures, delicate tissue handling, preservation of ovarian tissue, minimal electrocoagulation to prevent tissue necrosis and promote optimal healing, precise restoration of normal anatomy, and prevention of adhesions. In this study, the overall rate of spontaneous pregnancy after reproductive surgery is relatively high, achieving 51.7% (524/1013) in our institution and 50.3% (98/195) in the external cohort, which are similar to the previous reports (3, 4, 26). Given that, more and more infertile patients younger than 38 years without absolute indication for IVF are willing to choose to diagnostic or operative laparoscopy combined with hysteroscopy in our hospital. Nonetheless, there is still significant variation regarding the pregnancy result for women desiring to get pregnant following reproductive surgery, which makes it especially challenging to evaluate the prognosis. Therefore, the individualized prediction of the postoperative pregnancy probability has become increasingly important in the era of precision medicine. The nomogram developed in this study represents a pioneering effort to visualize patients’ probability of achieving pregnancy in the postoperative 2-year period using machine learning algorithms, and serves as a reference for clinicians and infertile couples to help them with personalized decision-making about the mode of subsequent conception, natural or IVF.

The limitations of our study include its retrospective design, which may introduce some inevitable bias, and the fact that the training and validation cohorts were ethnically homogeneous and limited to East China. Therefore, it is important to validate our data longitudinally in a more ethnically diverse patient population. In addition, unlike IVF’s timely feedback outcome (2–3 weeks), pregnancy rates after surgery lack continuous tracing because of the longer expectation period for spontaneous conception. Therefore, the pregnancy outcomes after reproductive surgery were followed only after 2-year at our department, leading to hard to perform survival analysis. In future updates, we will attempt to perform larger, multicenter, prospective studies and analyze long-term follow-up survival data. Third, if women with infertility undergo laparoscopy, it is clinical routine to perform hysteroscopy concurrently to rule out any concurrent endometrial abnormality at our institution. Hence, we were unable to include hysteroscopy as a covariate in the multivariable model. Last, it is the wide heterogeneity of the principle and skill of reproductive surgery in different medical institutions that poses the greatest challenge to the extrapolation capacity of the model.

## Conclusions

The first user-friendly web-based nomogram with good predictive ability was proposed in the current study to timely detect the possibility of natural conception after reproductive surgery. The model can be widely applied into the clinical practice and help guide clinicians and infertile couples make sensible decision of choosing the mode of subsequent conception, natural or IVF, to further improve the reproductive health in the population level. However, cross-institutional large-cohort prospective studies are needed to verify our model.

## Data availability statement

The original contributions presented in the study are included in the article/**Supplementary Material**. Further inquiries can be directed to the corresponding authors.

## Ethics statement

The studies involving humans were approved by the Ethics Committees of Yantai Yuhuangding Hospital (YT2023-054) and Linyi People’s Hospital [LYRMYY (2023-04-036)]. The studies were conducted in accordance with the local legislation and institutional requirements. The ethics committee/institutional review board waived the requirement of written informed consent for participation from the participants or the participants’ legal guardians/next of kin because this research was a retrospective cohort study, the data was made anonymous and there was no need for informed consent.

## Author contributions

ZL: Conceptualization, Data curation, Formal analysis, Funding acquisition, Investigation, Software, Visualization, Writing – original draft, Writing – review & editing. MW: Data curation, Formal analysis, Investigation, Methodology, Resources, Software, Visualization, Writing – original draft, Writing – review & editing. SH: Data curation, Formal analysis, Methodology, Project administration, Resources, Validation, Visualization, Writing – original draft, Writing – review & editing. XW: Conceptualization, Formal analysis, Funding acquisition, Investigation, Methodology, Software, Validation, Writing – original draft, Writing – review & editing. XL: Formal analysis, Validation, Visualization, Writing – original draft, Writing – review & editing. XX: Investigation, Methodology, Project administration, Supervision, Validation, Writing – original draft, Writing – review & editing. HB: Investigation, Methodology, Project administration, Supervision, Validation, Writing – original draft, Writing – review & editing.
