# Supplementary material for: Derivation and validation of the first web-based nomogram to predict the spontaneous pregnancy after reproductive surgery using machine learning models
Source: Front Endocrinol (Lausanne). 2024 Jul 2;15:1378157. doi: 10.3389/fendo.2024.1378157 (PMC11250273; doi:10.3389/fendo.2024.1378157)
Supplement: Supplementary file 1 [file Table_1.docx]

**Additional file 1**

[Table S1. Description of performed reproductive surgical procedures in infertile patients. 2](#_Toc155624737)

[Table S2. General characteristics of the training cohort before and after deletion of missing values. 4](#_Toc155624738)

[Table S3. The coefficients of Lasso regression analysis. 6](#_Toc155624739)

[Table S4. Net benefits for different threshold probabilities in the training (A), internal (B) and external (C) validation cohort. 7](#_Toc155624740)

**Table S1. Description of performed reproductive surgical procedures in infertile patients.**

| **Main operative diagnoses** | **Laparoscopy^a^ (n)** | **Hysteroscopy (n)** |
| --- | --- | --- |
| diagnostic surgery (150) | Diagnostic laparoscopy (117) | Diagnostic (122) |
|  | Removal of subserous or intraligamentary fibroids (23) | Resection of small/large septum (7/3) |
|  | Removal of unilateral ovarian cysts (excluding endometriomas) (10) | Other operative hysteroscopy^b^ (18) |
| Endometriosis mild-moderate (r-ARSM stage I-II) (196) | Electrocoagulation or excision of peritoneal endometriosis (189) | Diagnostic (138) |
|  | Enucleation of endometrioma <3 cm (42) | Resection of small/large septum (13/7) |
|  |  | Other operative hysteroscopy^b^ (38) |
| Endometriosis severe (r-ARSM stage III-IV) (100) | Removal of unilateral or bilateral endometrioma (88) | Diagnostic (45) |
|  | Salpingo ovariolysis due to endometriotic adhesions (45) | Resection of small/large septum (9/6) |
|  | Excision of endometriotic nodule in the bladder (22) | Other operative hysteroscopy^b^ (40) |
|  | Excision of endometriotic nodule from sacrouterine ligaments (64) |  |
|  | Deep infiltrating endometriosis (5) |  |
| Intramural fibroids (20) | Removal of single intramural fibroid between 2–8 cm (8) | Diagnostic (12) |
|  | Removal of multiple intramural fibroids (12) | Resection of small/large septum (2/0) |
|  |  | Other operative hysteroscopy^b^ (6) |
| Tubal factor_unilateral (192)^c^ | Unilateral neostomy and eversion with sutures (185) or with bipolar electrocoagulation (4) | Diagnostic (142) |
|  | Unilateral salpingectomy (22) | Resection of small/large septum (12/5) |
|  | Previous salpingectomy due to ectopic pregnancy (14) | Other operative hysteroscopy^b^ (33) |
| Tubal factor_bilateral (213) ^c^ | Bilateral neostomy with sutures (56) | Diagnostic (173) |
|  | Unilateral salpingectomy and contralateral neostomy (157) | Resection of small/large septum (3/3) |
|  |  | Other operative hysteroscopy^b^ (34) |
| Miscellaneous (142) | Endometriosis and additional intramural or submucous fibroids (26) | Diagnostic (111) |
|  | Endometriosis and severe damage of at least one tube (76) | Resection of small/large septum (5/2) |
|  | Severe adhesions of one tube and additional intramural or submucous fibroids (42) | Other operative hysteroscopy^b^ (24) |

^a^ Some women had more than one pathology found and corrected at laparoscopy.

^b^ Other operative hysteroscopy: removal of submucous fibroids, polyps or synechiae.

^c^ Retaining at least one roughly normal fallopian tube.

r-ARSM=the revised American Society for Reproductive medicine

**Table S2. General characteristics of the training cohort before and after deletion of missing values.**

| **Variables** | **Before deleting the missing value** | **After deleting the missing value** | **Missing cases*** | ***P*-value overall** |
| --- | --- | --- | --- | --- |
|  | ***N=1043*** | ***N=1013*** | ***N=30*** |  |
| Pregnancy^a^: |  |  |  | 0.718 |
| No | 505 (48.4%) | 489 (48.3%) | 16 (53.3%) |  |
| Yes | 538 (51.6%) | 524 (51.7%) | 14 (46.7%) |  |
| Age (years)^b^ | 31.2 (3.36) | 31.2 (3.34) | 30.5 (3.99) | 0.302 |
| BMI (kg/m^2^)^b^: |  |  |  | 0.360 |
| 20~24.9 | 589 (56.5%) | 569 (56.2%) | 20 (66.7%) |  |
| <20 | 208 (19.9%) | 204 (20.1%) | 4 (13.3%) |  |
| 25~29.9 | 209 (20.0%) | 205 (20.2%) | 4 (13.3%) |  |
| ≥30 | 37 (3.55%) | 35 (3.46%) | 2 (6.67%) |  |
| Infertility_type^a^: |  |  |  | 0.891 |
| primary | 526 (50.4%) | 510 (50.3%) | 16 (53.3%) |  |
| secondary | 517 (49.6%) | 503 (49.7%) | 14 (46.7%) |  |
| Duration of infertility (years)^b^ | 2.59 (1.37) | 2.59 (1.36) | 2.58 (1.63) | 0.991 |
| Previous_pelvic_surgery^a^: |  |  |  | 0.522 |
| no | 1018 (97.6%) | 989 (97.6%) | 29 (96.7%) |  |
| yes | 25 (2.40%) | 24 (2.37%) | 1 (3.33%) |  |
| HSG^a^: |  |  |  | 0.673 |
| mild | 430 (41.7%) | 422 (41.7%) | 8 (47.1%) |  |
| moderate | 308 (29.9%) | 302 (29.8%) | 6 (35.3%) |  |
| severe | 292 (28.3%) | 289 (28.5%) | 3 (17.6%) |  |
| Surgical_procedures^a^: |  |  |  | 0.299 |
| diagnostic | 153 (14.7%) | 150 (14.8%) | 3 (10.0%) |  |
| endometriosis_mild_moderate | 203 (19.5%) | 196 (19.3%) | 7 (23.3%) |  |
| endometriosis_severe | 102 (9.78%) | 100 (9.87%) | 2 (6.67%) |  |
| intramural_fibroids | 22 (2.11%) | 20 (1.97%) | 2 (6.67%) |  |
| tubal_factor_unilateral | 199 (19.1%) | 192 (19.0%) | 7 (23.3%) |  |
| tubal_factor_bilateral | 216 (20.7%) | 213 (21.0%) | 3 (10.0%) |  |
| miscellaneous | 148 (14.2%) | 142 (14.0%) | 6 (20.0%) |  |
| Ovulation_monitoring^a^: |  |  |  | 0.385 |
| no | 424 (40.7%) | 409 (40.4%) | 15 (50.0%) |  |
| yes | 619 (59.3%) | 604 (59.6%) | 15 (50.0%) |  |
| AMH^a^: |  |  |  | 0.272 |
| normal | 952 (92.7%) | 940 (92.8%) | 12 (85.7%) |  |
| low | 31 (3.02%) | 30 (2.96%) | 1 (7.14%) |  |
| high | 44 (4.28%) | 43 (4.24%) | 1 (7.14%) |  |
| CA125 (U/mL)^b^ | 24.4 (8.32) | 24.3 (8.29) | 26.2 (9.65) | 0.427 |
| TC (mmol/L)^b^ | 4.58 (0.86) | 4.57 (0.84) | 5.04 (1.65) | 0.257 |
| TG (mmol/L)^b^ | 1.19 (0.52) | 1.19 (0.50) | 1.53 (1.19) | 0.237 |
| HDLC (mmol/L)^b^ | 1.54 (0.40) | 1.54 (0.39) | 1.73 (0.70) | 0.189 |
| LDLC (mmol/L)^b^ | 2.59 (0.66) | 2.59 (0.63) | 2.93 (1.34) | 0.216 |
| ALT (U/L)^b^ | 25.8 (12.0) | 25.7 (11.9) | 28.3 (13.7) | 0.317 |
| AST (U/L)^b^ | 25.2 (8.49) | 25.1 (8.53) | 26.6 (6.76) | 0.246 |
| Cr (μmol/L)^b^ | 58.0 (10.4) | 57.9 (10.3) | 60.8 (11.8) | 0.201 |
| INS (uU/mL)^b^ | 13.8 (6.65) | 13.8 (6.64) | 14.8 (7.24) | 0.539 |
| Glu (mmol/L)^b^ | 5.04 (0.76) | 5.05 (0.76) | 4.92 (0.85) | 0.515 |

*The missing variables of cases were not completely consistent with each other.

^a^Categorical variables were expressed as frequency (percentage values), and differences among cohorts were determined using the chi-square (χ^2^), Fisher’s exact test or Kruskal-Wallis rank sum test, as appropriate. ^b^All values were mean (standard-deviation) and tested by analysis of variance (Gaussian distribution) or Kruskal-Wallis rank sum test (nonnormal distribution).

BMI=body mass index; HSG=hysterosalpingography; AMH=anti-Müllerian hormone; CA125=carbohydrate antigen 125; TC=total cholesterol; TG= triglyceride; HDLC=high-density lipoprotein; LDLC=low-density lipoprotein; ALT=alanine aminotransferase; AST=aspartate transaminase; Cr=creatinine; INS=fasting insulin; Glu=fasting glucose.

**Table S3. The coefficients of Lasso regression analysis.**

| **Variables** | **Coefficients** |
| --- | --- |
| Intercept | 5.17267484 |
| Age | -0.14112365 |
| Infertility type | 0.64953494 |
| Duration of infertility | -0.53695343 |
| Ovulation monitoring | 0.46405472 |
| Surgical_procedures |  |
| diagnostic | . |
| endometriosis_mild_moderate | 1.02782468 |
| endometriosis_severe | -0.46462732 |
| intramural_fibroids | . |
| tubal_factor_unilateral | 0.08344723 |
| tubal_factor_bilateral | . |
| miscellaneous | -0.45759954 |
| AMH |  |
| normal | -0.09935399 |
| low | . |
| high | . |

AMH=anti-Müllerian hormone

**Table S4. Net benefits for different threshold probabilities in the training (A),** **internal (B) and external (C) validation cohort.**

**(A) The training cohort**

| **thresholds** | **All** | **None** | **predictive model** |
| --- | --- | --- | --- |
| 0 | 0.513762 | 0 | 0.513762 |
| 0.01 | 0.511589 | 0 | 0.511675 |
| 0.02 | 0.505882 | 0 | 0.506731 |
| 0.03 | 0.501577 | 0 | 0.503343 |
| 0.04 | 0.496148 | 0 | 0.498209 |
| 0.05 | 0.489116 | 0 | 0.493547 |
| 0.06 | 0.485685 | 0 | 0.490417 |
| 0.07 | 0.475401 | 0 | 0.481957 |
| 0.08 | 0.470104 | 0 | 0.478067 |
| 0.09 | 0.46418 | 0 | 0.475028 |
| 0.1 | 0.457088 | 0 | 0.470555 |
| 0.11 | 0.451514 | 0 | 0.468027 |
| 0.12 | 0.444839 | 0 | 0.466015 |
| 0.13 | 0.438736 | 0 | 0.461237 |
| 0.14 | 0.432991 | 0 | 0.456193 |
| 0.15 | 0.427193 | 0 | 0.45266 |
| 0.16 | 0.419444 | 0 | 0.449654 |
| 0.17 | 0.411379 | 0 | 0.447559 |
| 0.18 | 0.403922 | 0 | 0.444039 |
| 0.19 | 0.400848 | 0 | 0.442883 |
| 0.2 | 0.390449 | 0 | 0.436895 |
| 0.21 | 0.380966 | 0 | 0.432342 |
| 0.22 | 0.373438 | 0 | 0.429212 |
| 0.23 | 0.36824 | 0 | 0.427801 |
| 0.24 | 0.356887 | 0 | 0.424266 |
| 0.25 | 0.352564 | 0 | 0.422958 |
| 0.26 | 0.339542 | 0 | 0.418265 |
| 0.27 | 0.33238 | 0 | 0.415074 |
| 0.28 | 0.325282 | 0 | 0.408637 |
| 0.29 | 0.313072 | 0 | 0.405651 |
| 0.3 | 0.305877 | 0 | 0.405935 |
| 0.31 | 0.292592 | 0 | 0.400332 |
| 0.32 | 0.28383 | 0 | 0.394284 |
| 0.33 | 0.269751 | 0 | 0.389294 |
| 0.34 | 0.25839 | 0 | 0.382725 |
| 0.35 | 0.252996 | 0 | 0.379527 |
| 0.36 | 0.236126 | 0 | 0.371313 |
| 0.37 | 0.222621 | 0 | 0.36662 |
| 0.38 | 0.210651 | 0 | 0.36176 |
| 0.39 | 0.197384 | 0 | 0.361705 |
| 0.4 | 0.186185 | 0 | 0.360333 |
| 0.41 | 0.169391 | 0 | 0.351411 |
| 0.42 | 0.154973 | 0 | 0.348344 |
| 0.43 | 0.140314 | 0 | 0.344878 |
| 0.44 | 0.129288 | 0 | 0.343681 |
| 0.45 | 0.122933 | 0 | 0.340715 |
| 0.46 | 0.0928 | 0 | 0.336778 |
| 0.47 | 0.081719 | 0 | 0.332469 |
| 0.48 | 0.05901 | 0 | 0.327106 |
| 0.49 | 0.038926 | 0 | 0.319765 |
| 0.5 | 0.019843 | 0 | 0.315676 |
| 0.51 | 0.003062 | 0 | 0.309399 |
| 0.52 | -0.0178 | 0 | 0.305456 |
| 0.53 | -0.04242 | 0 | 0.296537 |
| 0.54 | -0.06385 | 0 | 0.291184 |
| 0.55 | -0.08222 | 0 | 0.284819 |
| 0.56 | -0.11494 | 0 | 0.280714 |
| 0.57 | -0.13912 | 0 | 0.273846 |
| 0.58 | -0.15478 | 0 | 0.271478 |
| 0.59 | -0.1843 | 0 | 0.268735 |
| 0.6 | -0.22533 | 0 | 0.26083 |
| 0.61 | -0.25843 | 0 | 0.256747 |
| 0.62 | -0.2904 | 0 | 0.245768 |
| 0.63 | -0.3236 | 0 | 0.238652 |
| 0.64 | -0.36568 | 0 | 0.239444 |
| 0.65 | -0.40796 | 0 | 0.234307 |
| 0.66 | -0.43601 | 0 | 0.234584 |
| 0.67 | -0.48543 | 0 | 0.232609 |
| 0.68 | -0.53429 | 0 | 0.22409 |
| 0.69 | -0.58583 | 0 | 0.228258 |
| 0.7 | -0.63728 | 0 | 0.226751 |
| 0.71 | -0.70037 | 0 | 0.231783 |
| 0.72 | -0.76587 | 0 | 0.234392 |
| 0.73 | -0.82673 | 0 | 0.23318 |
| 0.74 | -0.89647 | 0 | 0.22379 |
| 0.75 | -0.96502 | 0 | 0.214822 |
| 0.76 | -1.05191 | 0 | 0.216755 |
| 0.77 | -1.12825 | 0 | 0.211249 |
| 0.78 | -1.22294 | 0 | 0.215218 |
| 0.79 | -1.35401 | 0 | 0.204346 |
| 0.8 | -1.48142 | 0 | 0.185814 |
| 0.81 | -1.62506 | 0 | 0.181705 |
| 0.82 | -1.77177 | 0 | 0.17915 |
| 0.83 | -1.93882 | 0 | 0.181917 |
| 0.84 | -2.13461 | 0 | 0.172045 |
| 0.85 | -2.32563 | 0 | 0.160686 |
| 0.86 | -2.59662 | 0 | 0.139592 |
| 0.87 | -2.81179 | 0 | 0.129563 |
| 0.88 | -3.19674 | 0 | 0.106617 |
| 0.89 | -3.59286 | 0 | 0.111366 |
| 0.9 | -4.05813 | 0 | 0.082231 |
| 0.91 | -4.64004 | 0 | 0.067926 |
| 0.92 | -5.16422 | 0 | 0.0734 |
| 0.93 | -6.38057 | 0 | 0.046698 |
| 0.94 | -7.73585 | 0 | 0.084302 |
| 0.95 | -9.62688 | 0 | 0.061983 |
| 0.96 | -12.7203 | 0 | 0.030571 |
| 0.97 | -16.7801 | 0 | 0.007601 |
| 0.98 | -19.4648 | 0 | -0.0128 |
| 0.99 | -35.947 | 0 | 0.025281 |
| 1 | -178.253 | 0 | 0.001404 |

**(B) The internal validation cohort**

| **thresholds** | **All** | **None** | **Predictive model** |
| --- | --- | --- | --- |
| 0 | 0.524839 | 0 | 0.524839 |
| 0.01 | 0.518706 | 0 | 0.519289 |
| 0.02 | 0.513618 | 0 | 0.515501 |
| 0.03 | 0.50979 | 0 | 0.512479 |
| 0.04 | 0.504353 | 0 | 0.50911 |
| 0.05 | 0.501096 | 0 | 0.507912 |
| 0.06 | 0.496048 | 0 | 0.501866 |
| 0.07 | 0.491357 | 0 | 0.499682 |
| 0.08 | 0.481591 | 0 | 0.496599 |
| 0.09 | 0.479334 | 0 | 0.495936 |
| 0.1 | 0.478352 | 0 | 0.492949 |
| 0.11 | 0.46867 | 0 | 0.48962 |
| 0.12 | 0.463124 | 0 | 0.485944 |
| 0.13 | 0.451596 | 0 | 0.484662 |
| 0.14 | 0.450424 | 0 | 0.482311 |
| 0.15 | 0.441719 | 0 | 0.478539 |
| 0.16 | 0.434072 | 0 | 0.471171 |
| 0.17 | 0.428532 | 0 | 0.46482 |
| 0.18 | 0.42791 | 0 | 0.458575 |
| 0.19 | 0.421852 | 0 | 0.456224 |
| 0.2 | 0.415393 | 0 | 0.452761 |
| 0.21 | 0.398577 | 0 | 0.4441 |
| 0.22 | 0.390447 | 0 | 0.443121 |
| 0.25 | 0.36841 | 0 | 0.434858 |
| 0.26 | 0.360749 | 0 | 0.43617 |
| 0.27 | 0.360616 | 0 | 0.432416 |
| 0.28 | 0.348094 | 0 | 0.429548 |
| 0.3 | 0.324858 | 0 | 0.422268 |
| 0.31 | 0.313539 | 0 | 0.420713 |
| 0.32 | 0.300304 | 0 | 0.421239 |
| 0.33 | 0.299911 | 0 | 0.416465 |
| 0.34 | 0.295675 | 0 | 0.41283 |
| 0.35 | 0.280069 | 0 | 0.409443 |
| 0.36 | 0.264554 | 0 | 0.407543 |
| 0.37 | 0.252988 | 0 | 0.403504 |
| 0.38 | 0.23071 | 0 | 0.402287 |
| 0.39 | 0.219832 | 0 | 0.391192 |
| 0.41 | 0.217315 | 0 | 0.381615 |
| 0.42 | 0.184947 | 0 | 0.377302 |
| 0.43 | 0.168925 | 0 | 0.371958 |
| 0.44 | 0.153168 | 0 | 0.364088 |
| 0.45 | 0.152866 | 0 | 0.356231 |
| 0.46 | 0.134337 | 0 | 0.356198 |
| 0.47 | 0.105135 | 0 | 0.34524 |
| 0.49 | 0.066948 | 0 | 0.327838 |
| 0.5 | 0.059326 | 0 | 0.330484 |
| 0.51 | 0.056684 | 0 | 0.32618 |
| 0.52 | 0.010746 | 0 | 0.322484 |
| 0.53 | -0.01168 | 0 | 0.322225 |
| 0.54 | -0.03214 | 0 | 0.330928 |
| 0.55 | -0.0336 | 0 | 0.330875 |
| 0.57 | -0.05719 | 0 | 0.326197 |
| 0.58 | -0.11839 | 0 | 0.315538 |
| 0.59 | -0.14508 | 0 | 0.304167 |
| 0.61 | -0.22252 | 0 | 0.305941 |
| 0.63 | -0.27993 | 0 | 0.290794 |
| 0.64 | -0.32547 | 0 | 0.294335 |
| 0.65 | -0.33037 | 0 | 0.283469 |
| 0.66 | -0.39622 | 0 | 0.29138 |
| 0.67 | -0.44147 | 0 | 0.269639 |
| 0.68 | -0.48388 | 0 | 0.254943 |
| 0.69 | -0.51891 | 0 | 0.255068 |
| 0.7 | -0.59617 | 0 | 0.241779 |
| 0.71 | -0.60254 | 0 | 0.232377 |
| 0.72 | -0.66465 | 0 | 0.228217 |
| 0.73 | -0.78219 | 0 | 0.203532 |
| 0.74 | -0.81326 | 0 | 0.18956 |
| 0.75 | -0.92481 | 0 | 0.179772 |
| 0.76 | -0.96234 | 0 | 0.169676 |
| 0.77 | -0.97078 | 0 | 0.172173 |
| 0.78 | -1.12802 | 0 | 0.171568 |
| 0.79 | -1.17493 | 0 | 0.159018 |
| 0.8 | -1.4009 | 0 | 0.16099 |
| 0.81 | -1.52305 | 0 | 0.130274 |
| 0.82 | -1.69827 | 0 | 0.112518 |
| 0.83 | -1.81809 | 0 | 0.109941 |
| 0.84 | -2.01031 | 0 | 0.092026 |
| 0.85 | -2.16878 | 0 | 0.064266 |
| 0.86 | -2.32325 | 0 | 0.052285 |
| 0.87 | -2.7342 | 0 | 0.010586 |
| 0.88 | -3.05651 | 0 | 0.022153 |
| 0.89 | -3.27495 | 0 | -0.00319 |
| 0.9 | -3.61932 | 0 | -0.05199 |
| 0.91 | -3.93055 | 0 | -0.07301 |
| 0.92 | -5.22968 | 0 | -0.06998 |
| 0.93 | -5.27711 | 0 | -0.07566 |
| 0.94 | -7.279 | 0 | -0.00591 |
| 0.95 | -9.18354 | 0 | -0.04512 |
| 0.96 | -11.7822 | 0 | -0.01346 |
| 0.97 | -16.2999 | 0 | 0.046512 |
| 0.98 | -21.1195 | 0 | 0.026578 |
| 0.99 | -26.4444 | 0 | 0.023256 |

**(C) The external validation cohort**

| **thresholds** | **All** | **None** | **Predictive model** |
| --- | --- | --- | --- |
| 0 | 0.502193 | 0 | 0.501458 |
| 0.01 | 0.499639 | 0 | 0.498213 |
| 0.02 | 0.492236 | 0 | 0.492343 |
| 0.03 | 0.488386 | 0 | 0.490548 |
| 0.04 | 0.484674 | 0 | 0.487256 |
| 0.05 | 0.477912 | 0 | 0.482726 |
| 0.06 | 0.474282 | 0 | 0.480008 |
| 0.07 | 0.466379 | 0 | 0.473484 |
| 0.08 | 0.465854 | 0 | 0.465632 |
| 0.09 | 0.452315 | 0 | 0.460612 |
| 0.1 | 0.451705 | 0 | 0.459941 |
| 0.11 | 0.449351 | 0 | 0.45483 |
| 0.12 | 0.434662 | 0 | 0.453733 |
| 0.13 | 0.432665 | 0 | 0.444353 |
| 0.16 | 0.42943 | 0 | 0.433649 |
| 0.17 | 0.406748 | 0 | 0.42588 |
| 0.2 | 0.376049 | 0 | 0.408454 |
| 0.21 | 0.373949 | 0 | 0.40385 |
| 0.22 | 0.365505 | 0 | 0.406046 |
| 0.23 | 0.359145 | 0 | 0.405598 |
| 0.25 | 0.340916 | 0 | 0.402511 |
| 0.26 | 0.327209 | 0 | 0.393678 |
| 0.33 | 0.261733 | 0 | 0.374086 |
| 0.34 | 0.260582 | 0 | 0.372105 |
| 0.4 | 0.175843 | 0 | 0.345413 |
| 0.41 | 0.166966 | 0 | 0.339622 |
| 0.42 | 0.147682 | 0 | 0.333053 |
| 0.43 | 0.126597 | 0 | 0.325705 |
| 0.44 | 0.110132 | 0 | 0.319078 |
| 0.48 | 0.050913 | 0 | 0.302431 |
| 0.5 | 0.013548 | 0 | 0.303902 |
| 0.52 | 0.011616 | 0 | 0.300036 |
| 0.54 | -0.03396 | 0 | 0.28615 |
| 0.55 | -0.07867 | 0 | 0.274937 |
| 0.56 | -0.12019 | 0 | 0.272878 |
| 0.57 | -0.166 | 0 | 0.262161 |
| 0.59 | -0.16953 | 0 | 0.257372 |
| 0.6 | -0.20667 | 0 | 0.260928 |
| 0.61 | -0.23095 | 0 | 0.248249 |
| 0.63 | -0.35292 | 0 | 0.254277 |
| 0.64 | -0.36763 | 0 | 0.270289 |
| 0.65 | -0.3745 | 0 | 0.264741 |
| 0.68 | -0.57268 | 0 | 0.253952 |
| 0.69 | -0.57551 | 0 | 0.243169 |
| 0.7 | -0.64798 | 0 | 0.242 |
| 0.71 | -0.69499 | 0 | 0.253936 |
| 0.72 | -0.73912 | 0 | 0.253107 |
| 0.75 | -0.96941 | 0 | 0.231418 |
| 0.76 | -0.99258 | 0 | 0.248467 |
| 0.78 | -1.08076 | 0 | 0.261696 |
| 0.8 | -1.52843 | 0 | 0.244922 |
| 0.81 | -1.54693 | 0 | 0.239628 |
| 0.82 | -1.69227 | 0 | 0.254177 |
| 0.83 | -1.82751 | 0 | 0.231947 |
| 0.86 | -2.54208 | 0 | 0.2146 |
| 0.87 | -2.68305 | 0 | 0.20271 |
| 0.88 | -2.71665 | 0 | 0.191426 |
| 0.89 | -3.63232 | 0 | 0.16746 |
| 0.91 | -4.72178 | 0 | 0.135743 |
| 0.92 | -5.09498 | 0 | 0.116576 |
| 0.93 | -5.61742 | 0 | 0.054666 |
| 0.94 | -6.76198 | 0 | 0.037861 |
| 0.96 | -10.1756 | 0 | -0.01881 |
| 0.97 | -14.233 | 0 | 0.076923 |
| 0.98 | -20.7982 | 0 | 0.041026 |
| 0.99 | -32.771 | 0 | 0.020513 |
